# Four Autophagy-Related lncRNAs Predict the Prognosis of HCC through Coexpression and ceRNA Mechanism

**DOI:** 10.1155/2020/3801748

**Published:** 2020-10-09

**Authors:** Hao Wu, Tiantian Liu, Jianni Qi, Chengyong Qin, Qiang Zhu

**Affiliations:** ^1^Department of Gastroenterology, Shandong Provincial Hospital, Cheeloo College of Medicine, Shandong University, Jinan, 250021 Shandong, China; ^2^Shandong Provincial Engineering and Technological Research Center for Liver Diseases Prevention and Control, Shandong Provincial Hospital, Cheeloo College of Medicine, Shandong University, Jinan, 250021 Shandong, China; ^3^Department of Central Laboratory, Shandong Provincial Hospital, Cheeloo College of Medicine, Shandong University, Jinan, 250021 Shandong, China

## Abstract

Abnormally expressed long noncoding RNAs (lncRNAs) have been reported to affect the occurrence and progression of hepatocellular carcinoma (HCC) by modulating the autophagy axis. However, none of studies has explored the clinical significance of these autophagy-related lncRNAs in HCC comprehensively. In this study, the RNA-seq, miRNA-seq, and clinical data of normal and HCC patients from the TCGA database and autophagy genes from the Human Autophagy Database were extracted. Subsequently, we screened out 78 differentially expressed autophagy-related lncRNAs, and four prognostic-related lncRNAs (LUCAT1, AC099850.3, ZFPM2-AS1, and AC009005.1) were eventually used to develop the prognostic model. This signature could be regarded as an independent prognostic signature for HCC patients and has the highest prediction efficiency than other clinicopathological factors for the 1-, 3-, and 5-year survival (AUC = 0.764, 0.738, and 0.717, respectively). Additionally, regardless of whether the clinical information is complete for HCC patients, the autophagy-related lncRNA model shows a good predictive power for the overall survival. Importantly, the coexpression network of 4 lncRNAs and 11 autophagy-related genes was constructed. Moreover, based on the bioinformatic analyses, our results found that LUCAT1 and ZFPM2-AS1 may affect the autophagic activity in HCC through the hsa-miR-495-3p/DLC1 and hsa-miR-515-5p/DAPK2 axis, respectively. In conclusion, we establish an effective prognostic model for HCC patients and shed new light on the autophagy-related regulatory mechanisms of the identified lncRNAs.

## 1. Introduction

Hepatocellular carcinoma (HCC) is the most frequent liver tumor, accounting for 75–80% of all primary liver cancer cases and arises from chronic liver inflammation and liver fibrosis mostly [1, 2]. Despite the major progress in risk factors, early diagnosis, and treatment techniques for HCC, the poor prognosis of HCC patients remains unsatisfactory (overall mortality to incidence rate, 0.95) [2]. Because of the complex molecular mechanisms and high cellular heterogeneity of HCC patients, traditional clinical parameters including AFP, TNM stage, and vascular invasion have the limited predictive power. Therefore, new and more accurate methods with a better understanding of the underlying HCC development mechanisms are urgently needed to facilitate early detection, help prognostic prediction, and guide individualized treatment.

Autophagy is a key intracellular process for degradation of damaged or unwanted protein and dysfunctional organelles, which is vital to maintain cellular homeostasis, metabolism, and survival [3, 4]. Dysregulation of the autophagic process has been reported to regulate a variety of pathological conditions and cancer development, including HCC [5–7]. The function of autophagy in HCC is a hotspot, and the autophagy process can play either a protective or a detrimental role in the occurrence and development of HCC depending on its activation status and different cellular conditions [7, 8]. For example, Wu et al. identified that the high expression of autophagic LC3B positively correlated with malignant progression and might be a prognostic biomarker for HCC [9]. In addition, growing research has demonstrated that autophagy-related gene signatures can act as a kind of new emerging biomarkers to robustly predict clinical outcomes in various types of cancers including HCC [10–12]. Therefore, exploring the mechanism of regulating autophagy in the tumorigenesis, metastasis, and treatment of HCC could contribute to the study of new therapeutic strategies and prognostic biomarkers for HCC patients. Studies have shown that autophagy is regulated by various factors. Except for classic energy signal molecules, protooncogenes, and suppressor genes, noncoding RNAs also play an important role [13–15].

Noncoding RNA refers to RNA that does not encode proteins. Among them, those with a length greater than 200 nucleotides are called “long noncoding RNA (lncRNA),” and the length less than 200 nucleotides is called “small noncoding RNA (sncRNA),” such as microRNA (miRNA) [16]. Unlike sncRNAs, the length of lncRNA allows it to regulate gene expression levels in a more complex transcription and translation network. Increasingly, recent studies have shown that lncRNAs can modulate autophagy effector molecules and pathways at different autophagic stages in HCC [15, 17–19]. However, the lncRNAs and their role in the autophagy axis in the prognosis of HCC are still under investigation. And the clinical role, particularly the prognostic role of autophagy-related lncRNAs in HCC, has yet to be determined.

In this study, we established an effective autophagy-related lncRNA signature for predicting the survival of HCC patients. Additionally, we comprehensively explored the molecular mechanism by which these autophagy-related lncRNAs affect the progression of HCC by regulating autophagy. Our study provides a theoretical basis in the potential therapeutic target selection.

## 2. Materials and Methods

### 2.1. Data Source

The raw RNA-Seq data, miRNA-Seq data, and the corresponding clinical information of patients with HCC were obtained from The Cancer Genome Atlas (TCGA, https://cancergenome.nih.gov/) database, which consisted of 374 HCC tumor and 50 normal liver tissue specimens. The 222 autophagy genes were extracted from the Human Autophagy Database (HADb, http://autophagy.lu/clustering/index.html), containing a list of genes directly or indirectly involved in the autophagy process reported in literature.

### 2.2. Identification of Differentially Expressed lncRNAs and Autophagy-Related lncRNAs

We downloaded the *Homo sapiens* ensemble ID (https://www.ensembl.org/) of RNA to retrieve the required expression information from RNA-Seq data. The differentially expressed lncRNAs were calculated using the package “edgeR” from R by comparing the HCC group and normal liver tissues. Differentially expressed lncRNAs with an absolute log2 fold change (FC) ≥ 2 and an adjusted *P* value < 0.05 were filtered out for subsequent analysis. Subsequently, we used the Pearson correlation to calculate the correlation between the lncRNAs and these 222 autophagy-related genes. Finally, the differentially expressed lncRNAs with the correlation coefficient > 0.3 and *P* < 0.05 with the autophagy-related genes were filtered out to be the autophagy-related lncRNAs [20].

### 2.3. Construction of the Autophagy-Related lncRNA Prognostic Signature

Univariate Cox regression and Kaplan-Meier (K-M) analyses were used to screen out autophagy-related lncRNAs that are significantly correlated with the overall survival (OS) of patients with HCC. The autophagy-related lncRNAs with a *P* value < 0.05 by univariate analysis and K-M analysis were included in the multivariate regression Cox analysis. Subsequently, we used the stepwise selection of variables based on the Akaike information criterion to identify optimal independent prognostic autophagy-related lncRNAs and the most appropriate model. The prognosis signature was constructed based on a linear combination of the regression coefficient derived from the multivariate Cox regression model (*β*) multiplied with its expression level. The cut-off point for the risk score was identified with the median to stratify HCC patients into the high-risk group and the low-risk group. The survival differences between the high-risk and the low-risk group were compared by the log-rank test. The time-dependent receiver operating characteristic (ROC) curves for predicting OS were drawn, and area under the curve (AUC) values were generated using R with the survival ROC package.

### 2.4. Internal Validation

An internal validation was performed to validate the predictive performance of the present prognostic model. The validation dataset was constructed by drawing 370 HCC patients with known survival times in the TCGA database using the bootstrap resampling method, which was recommended for internal validation of the prognostic model [21, 22].

### 2.5. Clinical Samples

We collected thirty-seven tumor tissues from primary HCC patients in Shandong Provincial Hospital, Shandong University, Jinan, Shandong, China, from July 2016 to December 2016. The inclusion criteria were as follows: (1) patients > 18 years old, (2) patients with pathologically confirmed HCC, and (3) patients who underwent curative surgical resection. Patients were excluded if they had other tumors or had recurrent HCC. A total of 11 normal liver tissues were collected from the patients with hepatic trauma undergoing surgical treatment. All tissues were fresh-frozen in liquid nitrogen immediately following surgical resection and stored at -80°C. And all procedures were approved by the Ethics Committee of Shandong Provincial Hospital.

### 2.6. Cell Culture

HCC cell lines (Huh7, MHCC97-h, LM3, and Bel-7402) and the LO2 cell line, human immortalized normal hepatocyte, were obtained from the Cell Bank of the Chinese Academy of Sciences (Shanghai, China). The LO2 cell line and HCC cell lines were cultured using Dulbecco's modified Eagle's medium (DMEM, GibcoBRL, Grand Island, NY, USA) with 10% fetal bovine serum (GibcoBRL, Grand Island, NY, USA) and antibiotics (100 U/mL penicillin and 100 *μ*g/mL streptomycin, Gibco, Grand Island, NY, and Scotland, UK). The humidified incubator containing 5% CO_2_ at 37°C was used to culture cell lines.

### 2.7. Quantitative Real-Time Polymerase Chain Reaction (qRT-PCR)

Total RNA from human tissues and cultured cells were extracted using the TRIzol reagent (Takara, Shiga, Japan). cDNAs were then generated using a reverse transcription kit (Takara, Shiga, Japan), and the gene expression was determined with real-time-PCR using a SYBR Green PCR kit (Takara, Shiga, Japan) in accordance with the manufacturer's instructions. The PCR primers are listed as follows: GAPDH-F: 5′-ACCCA CTCCT CCACC TTTGAC-3′, GAPDH-R: 5′-TGTTG CTGTA GCCAA ATTCG TT-3′; AC099850.3-F: 5′-TCGCT ATGTT TCCCA GGCTG TATT-3′, AC099850.3-R: 5′-TGCCA AGGAA TCTCT GAAGT CCAT-3′; LUCAT1-F: 5′-GTGTC CAAAT GCTGT CCTCA TCTC-3′, LUCAT1-R: 5′-ATCCT CGGGT TGCCT CTGTT TA-3′; ZFPM2-AS1-F: 5′-TGGTG GTATT TCTGC TGTTC TC-3′, ZFPM2-AS1-R: 5′-GTTCC ATCTT CCTCC TTGTC TAC-3′; and AC009005.1-F: 5′-GGCAA ACATC TCTTG TCCAT CCT-3′, AC009005.1-R: 5′-CTCTC CGCAT ATCCC TCCTT CT-3′. The 2^-*ΔΔ*Ct^ method was conducted to calculate the lncRNA expression. The Student *t*-test was used to compare the expression level of each lncRNA betwe3en different groups.

### 2.8. Gene Set Enrichment Analysis

In order to explore the pathways that are affected in the high-risk group and low-risk group, gene set enrichment analysis (GSEA, http://software.broadinstitute.org/gsea/index.jsp, version 3.0) was performed. Firstly, differentially expressed mRNAs were filtered out between tumor and adjacent normal liver tissues (absolute logFC ≥ 1.0 and *P* < 0.05). Then, we tested whether the differentially expressed mRNAs were enriched in the high-risk group and low-risk group using GSEA. The hallmarks were calculated using a normalized enrichment score (NES) and false discovery rate (FDR). Pathways with NES > 1 and FDR < 0.01 were considered significant enriched functional pathways.

### 2.9. Construction of the Coexpression and ceRNA Network

Differently expressed autophagy-related genes with an absolute log2 FC ≥ 1 and an adjusted *P* value < 0.05 were filtered out for subsequent analysis. These genes that highly correlated with autophagy-related lncRNA were used to construct the coexpression network.

The lncRNA-miRNA interactions were predicted by the miRcode database (http://www.mircode.org/) and starBase (http://starbase.sysu.edu.cn/) containing putative miRNA target sites in the long noncoding transcriptome. Differently expressed autophagy-related genes targeted by matched miRNAs were retrieved from miRDB, TargetScan (http://www.targetscan.org/), and miRTarBase (http://mirtarbase.mbc.nctu.edu.tw/php/index.php). Cytoscape software (version 3.7.0) was used to visualize the network.

### 2.10. Statistical Analysis

Data was presented as the mean ± standard error of mean (SEM). Statistical analyses were performed using R language (version 3.5.), SPSS 25.0 software (SPSS Inc., Chicago, IL), or GraphPad Prism 7 (GraphPad Software, La Jolla, CA). *P* values < 0.05 were considered statistically significant.

## 3. Results

### 3.1. Differentially Expressed Autophagy-Related lncRNAs in HCC

**Figure 1:**
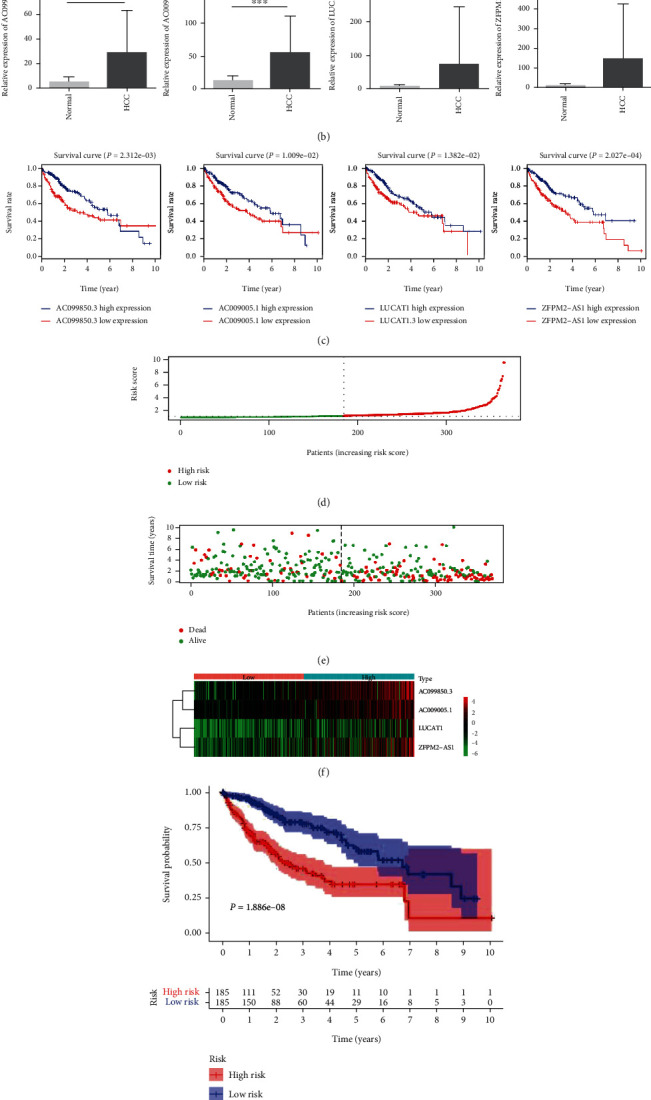
Construction of an autophagy-related lncRNA predictive model in HCC patients. (a) A heat map showing the differential expression of lncRNAs. (b) Differential expression of each lncRNA between normal and HCC liver tissues. ^∗^*P* < 0.05, ^∗∗^*P* < 0.01, and ^∗∗∗^*P* < 0.001. (c) K-M curves of OS of the four lncRNAs in HCC patients. (d) The distribution of the risk score. (e) Survival status of HCC patients in different groups. (f) A heat map showing the differential expression of each lncRNAs between the high-risk group and the low-risk group. (g) K-M curves of the autophagy-related lncRNA model for HCC patients. (h) ROC curves for the 1-, 3-, and 5-year survival prediction.

The RNA-Seq data of 374 HCC tissues and 50 normal liver tissues were obtained from the TCGA database. To retrieve the required lncRNA expression information using Homo sapiens' ensemble ID, the 14370 lncRNA expression profiles were included in the study. After differential expression analysis, 1097 differentially expressed lncRNAs were screened out by comparing HCC and normal liver tissues (∣logFC | ≥2.0, adjusted *P* < 0.05, Figure 1(a)). Furthermore, a total of 222 genes involved directly or indirectly in autophagy were downloaded via the online database HADb. The expression data of these autophagy-related genes were extracted from TCGA, which were used for further identifying their relationship with differentially expressed lncRNAs. Finally, 78 lncRNAs were selected according to correlation coefficient > 0.3 and *P* < 0.05 with autophagy-related genes, and these lncRNAs were regarded as autophagy-related lncRNAs.

### 3.2. Establishment and Internal Validation of an Autophagy-Related lncRNA Signature for the Prognosis of HCC Patients

Univariate Cox regression and K-M analyses based on 78 autophagy-related lncRNAs were used to screen prognostic biomarkers. A total of 9 autophagy-related lncRNAs which are identified as risk factors (HR > 1) and have prognostic value for HCC patients were screened out (Table 1). Furthermore, 4 independent prognostic autophagy-related lncRNAs (AC099850.3, LUCAT1, ZFPM2-AS1, and AC009005.1) were selected to develop the prognostic signature according to the multivariate Cox regression analysis based on the Akaike information criterion (Table 2). The expression levels and K-M curves for these lncRNAs were presented in Figures 1(b) and 1(c). Finally, the prognostic model was developed as follows: risk score = (0.125∗AC099850.3) + (0.109∗LUCAT1) + (0.055∗ZFPM2‐AS1) + (0.106∗AC009005.1). Based on the risk score, 370 HCC patients with survival times were classified as high-risk and low-risk groups according to the cut-off point. The distribution of the risk score and survival status of HCC patients is shown in Figures 1(d) and 1(e). The heat map of these four signature-related lncRNAs in the high-risk group and the low-risk group of HCC patients is displayed in Figure 1(f). K-M curves confirmed that the survival times of patients in the low-risk group were longer than those of patients in the high-risk group (2.636 ± 0.158 years vs. 1.753 ± 0.126 years, *P* < 0.0001, Figure 1(g)). ROC curves of OS were used to reveal the predictive performance of the four lncRNA risk signatures. The AUC values of the signature for the 1-, 3-, and 5-year survival were 0.765, 0.702, and 0.655, respectively (Figure 1(h)).

**Figure 2:**
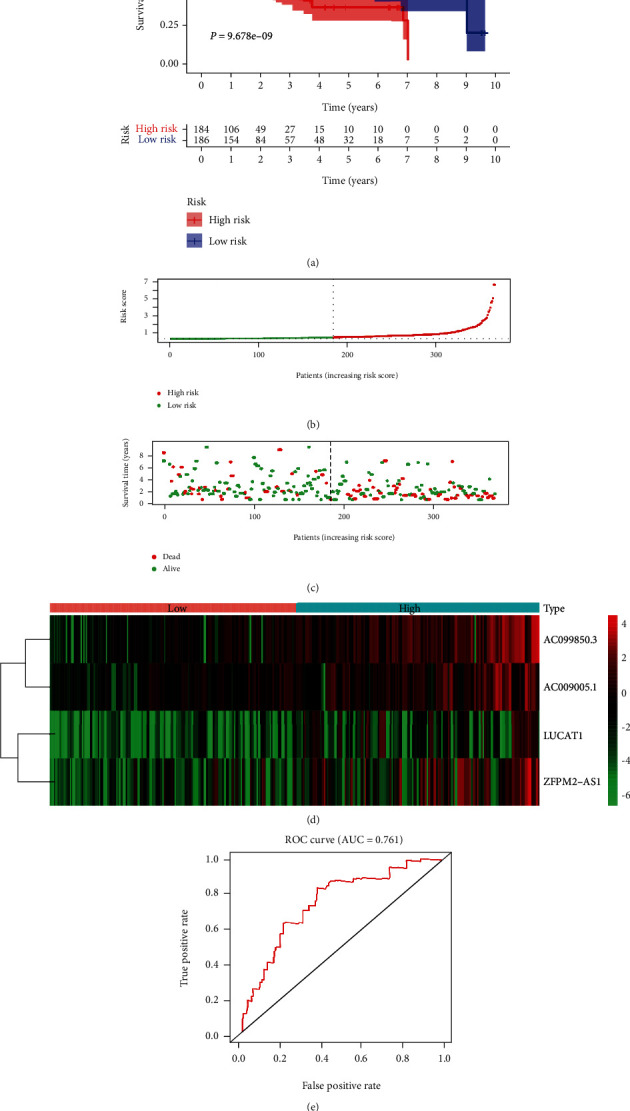
Validation of the autophagy-related lncRNA prognostic signature. (a) The survival curve of the model for the probability of OS in the validation of HCC cohort. (b) The distribution of the risk score in the validation cohort. (c) Survival status and survival time of HCC patients in the validation cohort. (d) A heat map showing the differential expression of each lncRNA. (e) ROC curve validates the prognostic significance of the signature in the validation cohort.

An internal validation cohort (*n* = 370) was assembled by random drawing with the replacement method from the model cohort (*n* = 370). Risk prediction scores for patients in the validation cohort was calculated. 370 patients in the validation cohort were stratified into the high-risk group (*n* = 185) and low-risk group (*n* = 185) following the median cut-off predicted value. The survival curve analysis indicated that the OS rate in the high-risk group was significantly poorer than that in the low-risk group (*P* = 9.672*e* − 09, Figure 2(a)). The distribution of the risk prediction score in the validation cohort is presented in Figure 2(b). The survival status and survival time in the validation cohort are presented in Figure 2(c). The heat map of these five signature-related lncRNAs in the high-risk and low-risk groups of HCC patients in the validation cohort is displayed in Figure 2(d). The AUC value of the signature was 0.761 (Figure 2(e)).

### 3.3. Molecular Pathways Disturbed between the High-Risk Group and the Low-Risk Group

**Figure 3:**
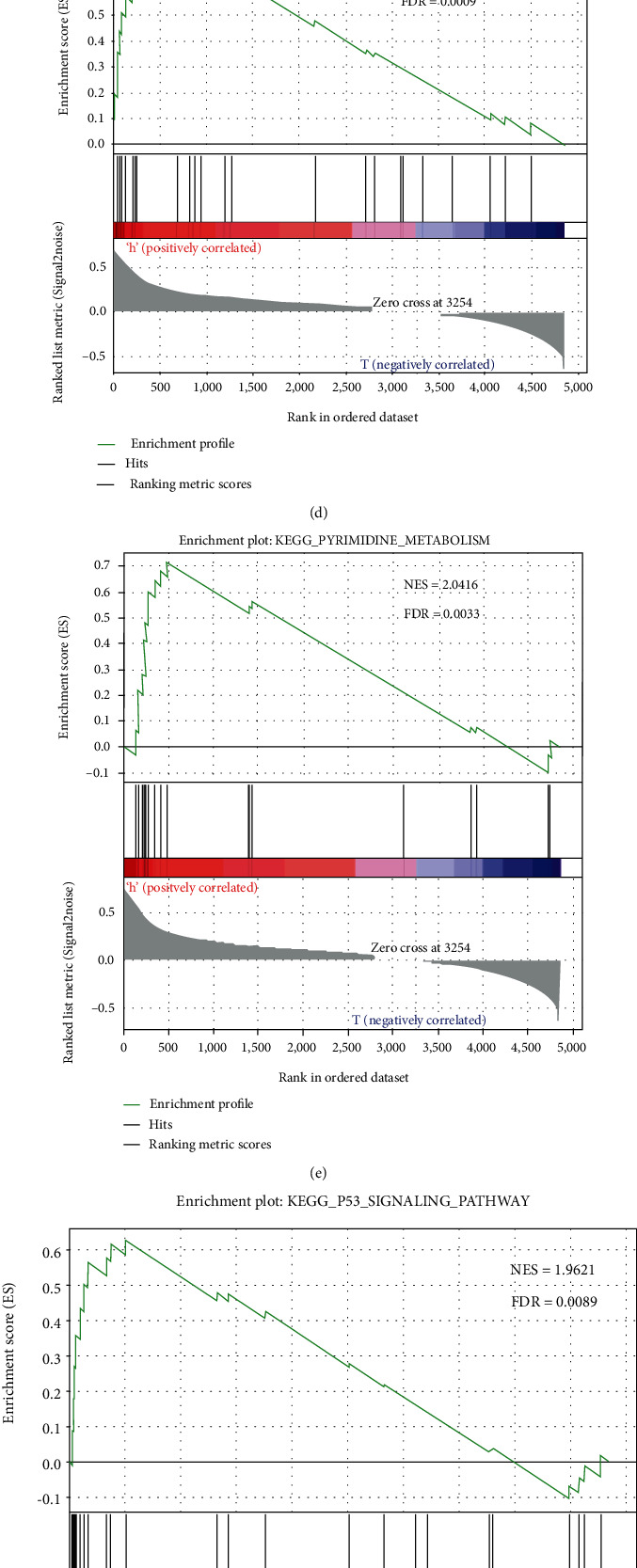
Gene set enrichment analysis. (a) Differentially expressed mRNAs. Red dots represent upregulated RNAs, and green dots represent downregulated RNAs. Gene set enrichment analysis indicated significant enrichment pathways in the high-risk group (b–f) and the low-risk group (g).

Using *P* < 0.05 and absolute logFC ≥ 1.0 as cut-offs, we found that 4851 mRNAs were differentially expressed between tumor and adjacent normal liver tissues (Figure 3(a)). Only these genes were included in the further study. GSEA was computed to pick up the molecular pathways disturbed between the high-risk group and the low-risk group. Only when the FDR < 0.01 were achieved could gene sets be considered significantly enriched. The results revealed that the “oocyte meiosis,” “cell cycle,” “progesterone-mediated oocyte,” “pyrimidine metabolism,” and “P53 signaling” pathways were enriched in the high-risk group (Figures 3(b)–3(f)). Genes coexpressed in the low-risk group were significantly enriched in the “PPAR signaling pathway” (Figure 3(g)). Several studies have indicated that these pathways were associated with the development of HCC. Taken together, the GSEA analyses implied that the four-autophagy-related lncRNA signature was associated with the HCC development and progression, which might provide strong evidence for a cancer-targeted treatment.

### 3.4. Relationship between the Four-Autophagy-Related lncRNA Model and Clinicopathological Features

After filtering out patients with incomplete clinical information, a total of 235 HCC patients were included in the analysis. Firstly, we determined the clinical value of the autophagy-related lncRNA signature regarding the age, gender, grade, and the tumor stage. Results showed that the signature was significantly associated with the grades (*P* = 0.017) and M and N stages (*P* < 0.001 and *P* = 0.007, respectively), suggesting that this lncRNA signature might be associated with the progression of HCC (Table 3).

**Figure 4:**
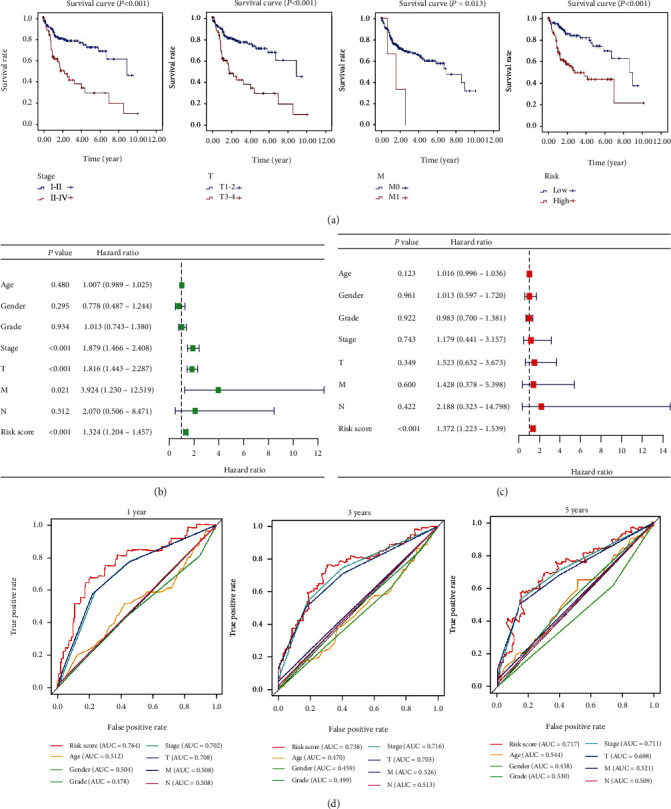
Relationship between the autophagy-related lncRNA prognostic signature and clinicopathological features of HCC. (a) K-M curves of stage, T stage, metastasis, and risk scores for the probability of OS in the HCC patients. The forest plot of univariate (b) and multivariate (c) Cox regression analyses in HCC patients. (d) ROC curves validate the prognostic significance of autophagy-related lncRNA prognostic indicators and clinicopathological features.

K-M curves showed that patients with high stage, high T stage, distant metastasis, or high-risk scores have worse prognosis (Figure 4(a)). Subsequently, we performed univariate and multivariate Cox regression analyses to verify the independent predictive value of the four lncRNA signatures for OS. The univariate Cox analysis showed that the tumor stage, T and N stages, and the autophagy-related lncRNA signature were all correlated with the survival of HCC patients (Figure 4(b)). Then, those factors were included in a multivariate Cox analysis, which showed only this signature to be an independent predictive factor (HR = 1.921, 95% CI = 1.013–3.644, *P* < 0.0001, Figure 4(c)). Thus, our results confirmed that the autophagy-related lncRNA signature could be used as an independent prognostic factor in clinical practice.

Furthermore, the predictive power value for survival of this signature and clinical factors for survival were compared using ROC curve analysis. The results suggested that the pathological stage and T stage show better prognostic ability for survival than the other factors. The AUCs of the pathological stage were 0.702, 0.716, and 0.711, respectively, for the 1-, 3-, and 5-year survival (Figure 4(d)). In addition, the AUC of the T stage was 0.708, 0.703, and 0.698 at the survival time of 1, 3, and 5 years, respectively. However, the autophagy-related lncRNA model shows the best favorable indicator for survival prediction in value in HCC patients than other clinicopathological factors for the 1-, 3-, and 5-year survival (AUC = 0.764, 0.738, and 0.717, respectively. Figure 4(d)).

### 3.5. Prognostic Value of Autophagy-Related lncRNA Signature in HCC Patients without Complete Clinical Information

**Figure 5:**
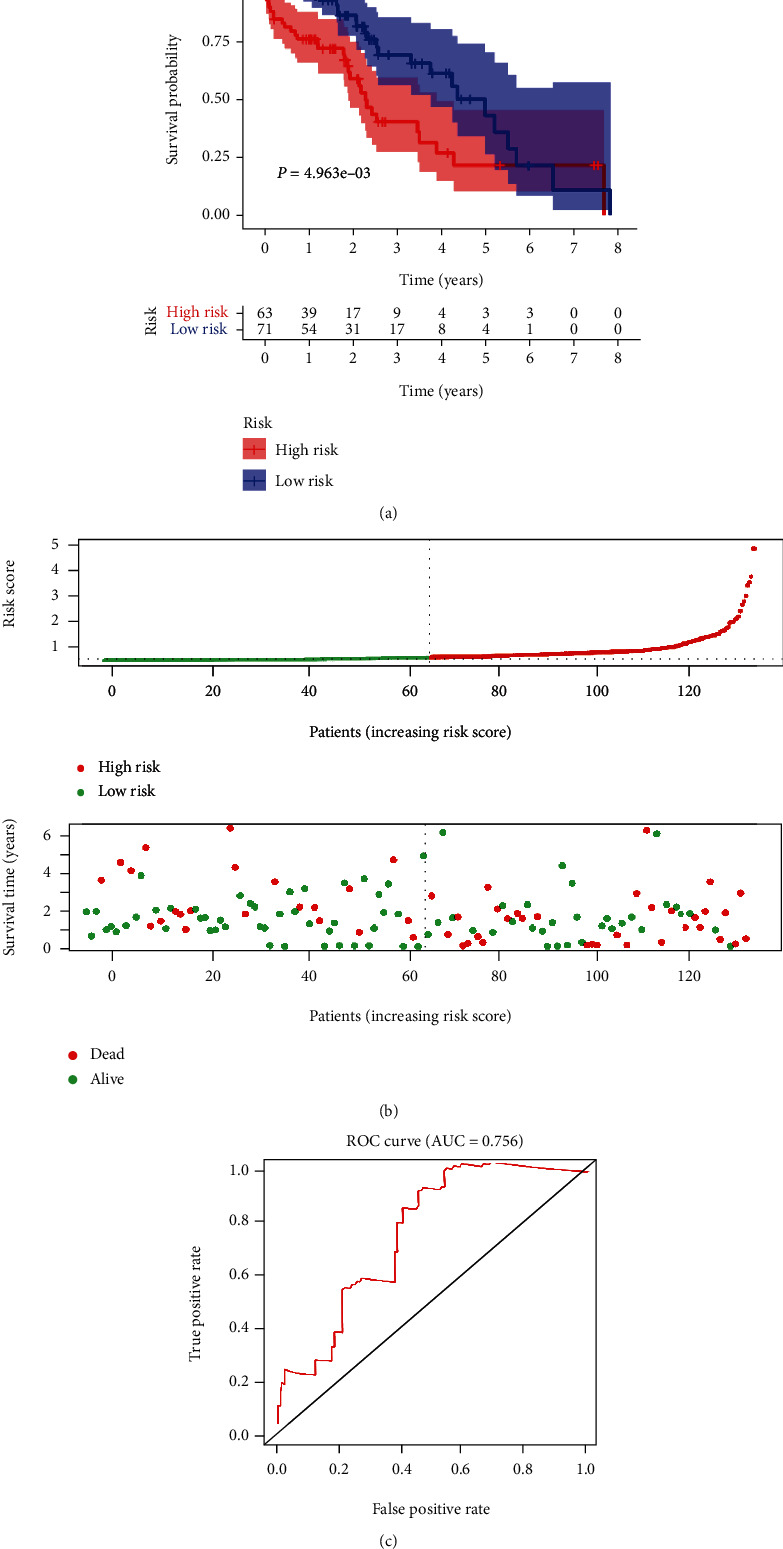
Prognostic value of the autophagy-related lncRNA signature in HCC patients without complete clinical information. (a) K-M curve of OS of the signature. (b) The distribution of the risk score and survival status of HCC patients with incomplete clinical information. (c) ROC curve for survival prediction.

We also included the other 136 patients with incomplete clinical information in the subsequent analysis. K-M curves confirmed that the survival times of patients in the low-risk group were longer than those of patients in the high-risk group (2.072 ± 0.181 years vs. 1.651 ± 0.196 years, *P* = 0.004963, Figure 5(a)). The distribution of the risk prediction score and survival status in this cohort .is presented in Figure 5(b). ROC curves of OS were used to reveal the predictive performance of the four-autophagy-related lncRNA risk model in HCC patients without complete clinical information. The AUC value of the signature for the 1-year survival was 0.756 (Figure 5(c)). Altogether, the results show that this risk score model also has good prediction efficiency in HCC patients with incomplete clinical information.

### 3.6. Validating the Expression Level of the Four lncRNAs in Clinical HCC Patients and *In Vitro*

**Figure 6:**
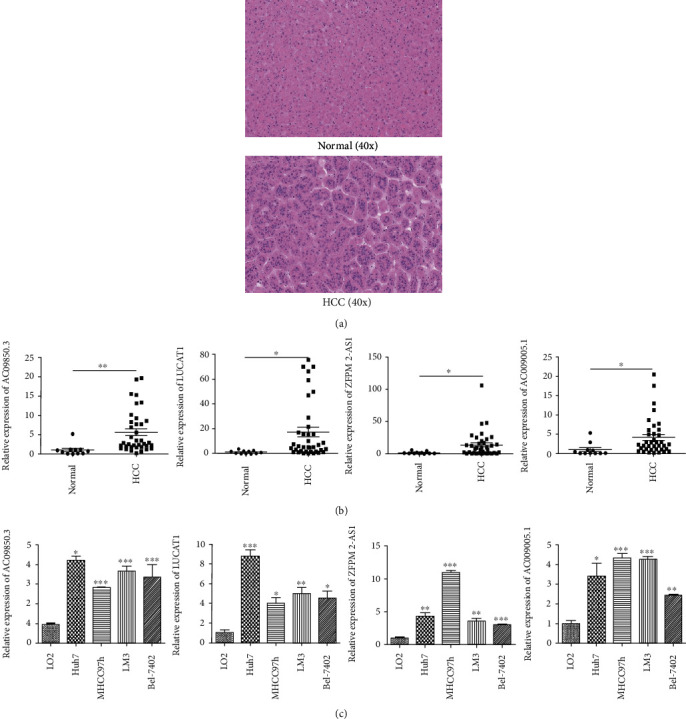
Validating the expression level of the four lncRNAs. (a) Hematoxylin-eosin staining shows that all patients were diagnosed as primary HCC (40x). (b) Differential expression of each lncRNA between normal liver samples and HCC tissues. (c) The expression level of each lncRNA in the normal hepatocyte cell line and HCC cell lines. ^∗^*P* < 0.05, ^∗∗^*P* < 0.01, and ^∗∗∗^*P* < 0.001.

AC099850.3, LUCAT1, ZFPM2-AS1, and AC009005.1 were highly expressed in tumor tissues than normal liver tissues according to the result of the TCGA database. Subsequently, we determined the expression levels of these four lncRNAs in 37 tumor tissues from primary HCC patients and 11 normal liver tissues using qRT-PCR. Hematoxylin-eosin staining was used to assess whether the tissue is normal or HCC (Figure 6(a)). As the results, all lncRNAs—AC099850.3, LUCAT1, ZFPM2-AS1, and AC009005.1—displayed high expression patterns in HCC tumor tissues when compared with normal samples (Figure 6(b)), which was consistent with the findings in the TCGA cohort.

Additionally, we detected the expression level of each lncRNA in LO2 and HCC cell lines (Huh7, MHCC97-h, LM3, and Bel-7402). All HCC cell lines indicated higher expression levels of each lncRNA compared to the normal hepatocyte cell line LO2 (Figure 6(c)).

### 3.7. Mechanism of Regulatory Network for the Four Autophagy-Related lncRNAs

**Figure 7:**
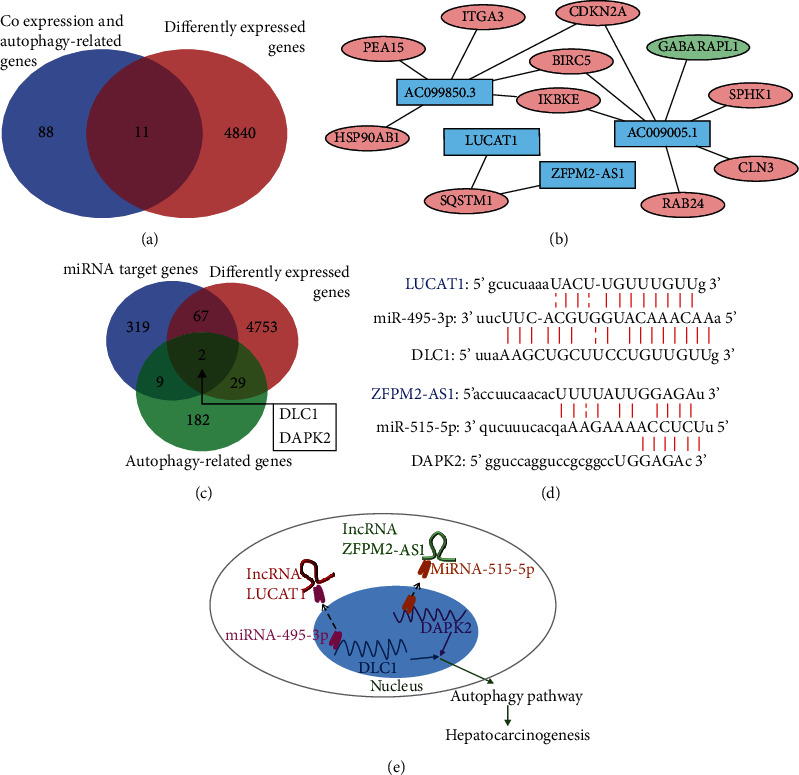
Construction of the regulatory network of the four autophagy-related lncRNAs. (a) Venn diagram analysis showing the genes that were differently expressed autophagy-related and having expression correlation with these four lncRNAs. (b) The coexpression networks of the four autophagy-related lncRNAs. The blue rectangles represent the lncRNAs. Red and green ellipses represent the upregulated and downregulated differentially expressed autophagy-related mRNAs, respectively. (c) Venn diagram analysis of the miRNA target genes. (d) The predictive binding site of the ceRNA network. (e) The schematic illustrates the mechanism by which LUCAT1 and ZFPM2-AS1 affect autophagy through the ceRNA mechanism to regulate HCC.

We found that 99 autophagy-related genes are related to the expression of lncRNAs (AC099850.3, LUCAT1, ZFPM2-AS1, and AC009005.1) according to correlation coefficient > 0.3 and *P* < 0.05 (Table S1). Among these 99 autophagy-related genes, only 11 genes were differentially expressed and selected to construct the coexpression networks (Figure 7(a)). The visualization of coexpression networks of the 4 lncRNAs and mRNAs is shown in Figure 7(b).

For further analysis of the mechanisms of these four prognostic lncRNAs, the ceRNA network was also considered. The target relationships between the four autophagy-related lncRNAs and miRNAs were assessed using the miRcode and starBase. The result showed that 22 miRNAs have the binding domains with LUCAT1, ZFPM2-AS1, and AC009005.1. Furthermore, we predicted the target mRNAs of these miRNAs through miRDB, miRtarBase, and TargetScan. A total of 367 mRNAs were filtered out for subsequent analysis. Lastly, DAPK2 and DLC1 were selected as the differently expressed and autophagy-related overlapping genes (Figure 7(c)). Finally, according to the above results, LUCAT1 and ZFPM2-AS1 can regulate the biological behavior through the ceRNA network. lncRNA LUCAT1 functioned as an autophagy promoter in HCC through sponging hsa-miR-495-3p (Figures 7(d) and 7(e)). What is more, ZFPM2-AS1 affects the autophagic activity in HCC through the hsa-miR-515-5p/DAPK2 axis (Figures 7(d) and 7(e)). In addition, we collect and analyze the expression levels of miR-495-3p/DLC1 and miR-515-5p/DAPK2 and the survival information of HCC patients based on the TCGA database. The univariate Cox regression and K-M analyses were presented to evaluate the prognostic value of miR-495-3p, DLC1, miR-515-5p, and DAPK2. As shown in Table S3, only DLC1 was correlated with the overall survival of patients with HCC.

## 4. Discussion

Autophagy is considered to play a crucial role in the occurrence and treatment of tumors. In recent years, as the understanding of lncRNA has gradually deepened, its role in the regulation of autophagy has also received increasing attention. Several studies have described the role of lncRNAs and autophagy in liver disease and particularly in HCC [23–27]. Therefore, it is important to understand the molecular pathogenesis mechanisms underlying the relationship between lncRNAs and autophagy in the initiation and development of HCC. Moreover, an increasing number of autophagy-related genes signatures serve as valuable prognostic signatures for tumor patients. However, none of the studies has comprehensive analysis of autophagy-related lncRNAs and explores its clinical significance in HCC. Here, we aimed to establish an autophagy-related lncRNA signature in HCC and explore the molecular mechanism of these lncRNAs and their role in the autophagy axis. Our study may lead to a better understanding of potential therapeutic approaches and biomarker assessment for HCC patients.

In this study, four lncRNAs, AC099850.3, LUCAT1, ZFPM2-AS1, and AC009005.1, were found to be significantly associated with autophagy-related genes and the survival of HCC patients and were selected to develop the prognostic model according to the TCGA and HADb databases. This signature has the highest prediction efficiency in the model cohort (AUC = 0.765) and in the validation cohort (AUC = 0.761) for 1-year OS, respectively. The lncRNA risk prediction score could stratify HCC patients into the low-risk group and high-risk group, and the OS rate of high-risk patients was significantly poorer than that of low-risk patients. Subsequently, we evaluated the clinical value of the autophagy-related lncRNA signature. Results showed that the model was significantly associated with the grade M and N stages, suggesting that this lncRNA signature might be associated with the progression of HCC. Our results also confirmed that the autophagy-related lncRNA risk score could be used as an independent prognostic factor in clinical practice according to the univariate and multivariate Cox regression analyses. Furthermore, ROC curve analysis suggested that the autophagy-related lncRNA model showed better predictive value in HCC patients than other clinicopathological factors. Importantly, we found that this risk score model also has good prediction efficiency in HCC patients with incomplete clinical information.

The Coding Potential Calculator (CPC, http://cpc.cbi.pku.edu.cn/) and Coding Potential Assessment Tool (CPAT, http://lilab.research.bcm.edu/cpat/index.php) were used to evaluate the coding ability of these lncRNAs [28, 29]. Table S2 showed that these lncRNAs (AC099850.3, LUCAT1, ZFPM2-AS1, and AC009005.1) were noncoding RNA. Subsequently, we analyze the regulatory network of the four autophagy-related lncRNAs comprehensively. Among these lncRNAs, LUCAT1 influences the proliferation, migration, and invasion of tumor cells, being involved in the cell cycle of many cancer cells [30–32]. It has been shown as novel players in predicting tumor recurrence and promotes tumorigenesis by inhibiting ANXA2 phosphorylation in HCC [33]. However, the role of LUCAT1 in the prognosis of HCC through autophagy remains unclear. In this study, we confirmed the expression level of LUCAT1 in HCC tissue samples and cell lines. Additionally, according to the coexpression analysis, our study found that LUCAT1 might promote the tumorigenesis of HCC by regulating autophagy via SQSTM1 (cor = 0.526, *P* < 0.0001). Furthermore, it has been reported that lncRNAs are able to regulate miRNAs through binding and separating them from their target mRNAs to affect the autophagic activity [34]. Few studies have reported the LUCAT1-related ceRNA regulatory mechanism. For example, Wang et al. found that LUCAT1 was critical for proliferation and invasion of ccRCC cells by inhibiting the expression of miR-495-3p, which subsequently regulated the expression of SATB1 [35]. However, in this study, we further constructed the LUCAT1-related ceRNA network, in which LUCAT1 regulated miR-495-3p through directly sponging it from the target DLC1 to affect the autophagic activity in HCC.

lncRNA ZFPM2-AS1 has been verified to be upregulated and plays tumor-promoting roles in human cancers [36–39]. For instance, lncRNA ZFPM2-AS1 promotes lung adenocarcinoma progression by interacting with UPF1 to destabilize ZFPM2 [40]. Recently, researchers reported that the cancer-promoting activities of ZFPM2-AS1 were mediated by the MIF–p53 signaling pathway in gastric cancer, by the miR-18b-5p–VMA21 axis in lung adenocarcinoma, by miR-137 in renal cell cancer, and by miRNA-511-3p and consequently increasing the FGFR2 expression in cervical cancer [36–39, 41]. ZFPM2-AS1 was previously identified as a prognostic lncRNA in a TCGA lncRNA-based prognostic signature investigation in HCC patient prognoses [42]. Additionally, Luo et al. identified that the expression levels of lncRNA ZFPM2-AS1 were significantly increased in HCC tissues compared with normal liver tissues, and higher expression levels of ZFPM2-AS1 were significantly associated with a less favorable prognosis of HCC [43], which were consistent with our finding. Nonetheless, none of these studies focus on the relationship between ZFPM2-AS1 and autophagy in cancers. In addition, the expression level and functions of ZFPM2-AS1 in HCC remain poorly understood. In this study, we explore the mechanisms of ZFPM2-AS1 in HCC. On one hand, bioinformatic analysis indicated that ZFPM2-AS1 might promote the tumorigenesis of HCC by regulating autophagy via SQSTM1. On the other hand, the ceRNA network is composed of ZFPM2-AS1, miR-515-5p, and DAPK2. ZFPM2-AS1 harbors a potential binding site for miR-515-5p. And the miR-515-5p has a potential binding site for the autophagy-related gene DAPK2.

Among these four autophagy-related lncRNA model, there is no report about the expression characteristics and related regulatory mechanisms of AC099850.3 and AC009005.1 in tumors. In the study, we used clinical specimens (HCC tissue samples and normal liver tissues) and cell lines (normal and HCC cell lines) for testing to confirm the expression level and stability of lncRNA AC099850.3 and AC009005.1, and the results were consistent with the findings in the TCGA cohort. Additionally, we identified the potential mechanisms of these lncRNAs in HCC using bioinformatic analysis. The results identified that AC099850.3 have the coexpression with the differently expressed autophagy-related genes, PEA15, IKBKE, CDKN2A, BIRC5, ITGA3, and HSP90AB1. In addition, AC009005.1 may be a novel oncogene in hepatocarcinogenesis by interacting with IKBKE, CDKN2A, BIRC5, SPHK1, RAB24, CLN3, and GABARAPL. Our results show that these two lncRNAs may influence the underlying mechanism of liver cancer development through the regulation of autophagy.

Although this identified risk score model is robust and promising, there are several limitations. We tried to search other databases to find an appropriate cohort for validation of our prediction model. However, we did not find a suitable dataset with expression profiles of all four lncRNAs and corresponding clinical data for survival analysis in the Gene Expression Omnibus (GEO) database and the International Cancer Genome Consortium (ICGC) data portal. In addition, our present study only validated the expression levels of the lncRNAs and conducted the bioinformatic analyses to provide a potential network of these 4 lncRNAs and two specific ceRNA mechanisms. However, comprehensive in vitro experiments need to be investigated to further verify the ceRNA regulation mechanism of LUCAT1/miR-495-3p/DLC1 and ZFPM2-AS1/miR-515-5p/DAPK2 and the coexpression networks of these lncRNAs.

## 5. Conclusions

In summary, our study has constructed a robust autophagy-related prognostic signature with four lncRNAs (LUCAT1, AC099850.3, ZFPM2-AS1, and AC009005.1) for survival prediction of HCC. Importantly, we have provided comprehensively regulatory mechanism understanding into the four lncRNAs and their role in the autophagy axis, which could be considered as prognostic biomarkers and contribute to the individual therapy research for HCC.

## Figures and Tables

**Table 1:** K-M and univariate Cox regression analyses of lncRNAs for OS of HCC patients.

lncRNA	KM	B	SE	HR	95% CI	*P* value
LUCAT1	0.013	0.164	0.038	1.179	1.095-1.269	<0.001
AC092171.2	0.005	0.077	0.032	1.080	1.015-1.149	0.016
MYLK-AS1	0.002	0.216	0.101	1.241	1.018-1.513	0.033
AC009005.1	0.010	0.152	0.042	1.165	1.073-1.264	<0.001
AC099850.3	0.002	0.135	0.024	1.145	1.093-1.199	<0.001
ZFPM2-AS1	<0.001	0.092	0.019	1.096	1.056-1.138	<0.001
AL606489.1	0.007	0.181	0.071	1.199	1.043-1.378	0.011
AC024361.1	0.038	0.308	0.137	1.360	1.040-1.779	0.025
LINC00942	0.014	0.036	0.008	1.037	1.021-1.053	<0.001

**Table 2:** The information of the 4 lncRNAs in the signature.

lncRNA	Ensemble ID	Chromosome	Coefficient^†^	HR^†^	95% CI^†^	*P* value^†^
AC099850.3	ENSG00000265415	Chr17q22	0.125	1.133	1.078-1.192	<0.001
LUCAT1	ENSG00000248323	Chr5q14.3	0.109	1.116	1.019-1.222	0.018
ZFPM2-AS1	ENSG00000251003	Chr8q23.1	0.055	1.056	1.010-1.104	0.016
AC009005.1	ENSG00000267751	Chr19p13.3	0.106	1.112	1.018-1.214	0.018

^†^Statistics derived from multivariate Cox proportional hazards regression analysis.

**Table 3:** Distribution of HCC patients' characteristics and the clinical correlation with the lncRNAs signature (*n* = 235).

Clinical parameter	Group	*n*	Risk score		
			Mean	SD	*P* value
Age	≤55	95	1.402	1.550	0.30946
	>55	140	1.208	1.241	
Gender	Female	74	1.218	1.348	0.60302
	Male	161	1.318	1.389	
Grade	G1-2	132	1.084	0.940	0.01671
	G3-4	103	1.546	1.754	
Stage	Stages I-II	163	1.200	1.377	0.14686
	Stages III-IV	72	1.482	1.356	
T	T1-2	167	1.193	1.362	0.10477
	T3-4	68	1.517	1.388	
M	M0	231	1.294	1.385	0.00021
	M1	4	0.835	0.12	
N	N0	231	1.293	1.385	0.00728
	N1	4	0.908	0.166	

## Data Availability

The data that support the findings of the study are available in The Cancer Genome Atlas database at https://cancergenome.nih.gov/ and the Human Autophagy Database at http://autophagy.lu/clustering/index.html.

## Supplementary Materials

Supplementary 1 Table S1: the relationship between the four lncRNAs and the differentially expressed autophagy-related mRNAs.

Supplementary 2 Table S2: the coding ability of the four lncRNAs according to the Coding Potential Calculator (CPC) and Coding Potential Assessment Tool (CPAT).

Supplementary 3 Table S3: K-M and univariate Cox regression analyses of RNAs for OS of 370 HCC patients.
