# Supplementary material for: Four Autophagy-Related lncRNAs Predict the Prognosis of HCC through Coexpression and ceRNA Mechanism
Source: Biomed Res Int. 2020 Oct 9;2020:3801748. doi: 10.1155/2020/3801748 (PMC7568797; doi:10.1155/2020/3801748)
Supplement: Supplementary 1 — Table S1: the relationship between the four lncRNAs and the differentially expressed autophagy-related mRNAs. [file 3801748.f1.docx]

**Table S1.** The relationship between the four lncRNAs and the differentially expressed autophagy-related mRNAs.

| lncRNA | mRNA | cor | *p*-value |
| --- | --- | --- | --- |
| AC099850.3 | BIRC5 | 0.698 | 5.36E-56 |
| AC099850.3 | CDKN2A | 0.450 | 4.42E-20 |
| AC099850.3 | HSP90AB1 | 0.376 | 5.42E-14 |
| AC099850.3 | IKBKE | 0.458 | 9.33E-21 |
| AC099850.3 | ITGA3 | 0.329 | 6.58E-11 |
| AC099850.3 | PEA15 | 0.445 | 1.51E-19 |
| LUCAT1 | SQSTM1 | 0.526 | 6.10E-28 |
| ZFPM2-AS1 | SQSTM1 | 0.557 | 8.19E-32 |
| AC009005.1 | BIRC5 | 0.392 | 3.29E-15 |
| AC009005.1 | CDKN2A | 0.311 | 8.37E-10 |
| AC009005.1 | CLN3 | 0.348 | 4.70E-12 |
| AC009005.1 | GABARAPL1 | -0.309 | 1.09E-09 |
| AC009005.1 | IKBKE | 0.426 | 6.43E-18 |
| AC009005.1 | RAB24 | 0.361 | 5.67E-13 |
| AC009005.1 | SPHK1 | 0.330 | 6.27E-11 |
