# Supplementary material for: Four Autophagy-Related lncRNAs Predict the Prognosis of HCC through Coexpression and ceRNA Mechanism
Source: Biomed Res Int. 2020 Oct 9;2020:3801748. doi: 10.1155/2020/3801748 (PMC7568797; doi:10.1155/2020/3801748)
Supplement: Supplementary 2 — Table S2: the coding ability of the four lncRNAs according to the Coding Potential Calculator (CPC) and Coding Potential Assessment Tool (CPAT). [file 3801748.f2.docx]

**Table S2.** The coding ability of the four lncRNAs according to Coding Potential Calculator (CPC) and Coding Potential Assessment Tool (CPAT).

| CPC |  |  |  |  |  |  |
| --- | --- | --- | --- | --- | --- | --- |
| GeneName | Sequence Name | Fickett  score | pI | ORF  integrity | Coding  probability | label |
| ZFPM2-AS1 | ENST00000524045.6 | 0.37045 | 9.996155 | 1 | 0.338683 | noncoding |
| AC009005.1 | ENST00000589457.2 | 0.44148 | 4.136902 | 1 | 0.022853 | noncoding |
| LINC00942 | ENST00000515614.3 | 0.29747 | 11.06976 | 1 | 0.099083 | noncoding |
| LUCAT1 | ENST00000513626.3 | 0.32386 | 11.08197 | 1 | 0.059223 | noncoding |
| AC099850.3 | ENST00000577660.1 | 0.41689 | 9.341370 | 1 | 0.219191 | noncoding |
| DDX11-AS1 | ENST00000500527.1 | 0.28779 | 4.926331 | 1 | 0.074238 | noncoding |

| CAPT |  |  |  |  |  |  |
| --- | --- | --- | --- | --- | --- | --- |
| GeneName | Sequence Name | ORF size | Ficket Score | Hexamer Score | Coding Probability | Coding Label |
| AC009005.1 | ENST00000589457.2 | 78 | 0.7855 | 0.060256 | 0.011756 | no |
| LINC00942 | ENST00000515614.3 | 315 | 0.5957 | 0.096308 | 0.089654 | no |
| ZFPM2_AS1 | ENST00000524045.6 | 345 | 0.5904 | -0.22709 | 0.016772 | no |
| LUCAT1 | ENST00000513626.3 | 231 | 0.7684 | -0.01390 | 0.030371 | no |
| AC099850.3 | ENST00000577660 | 258 | 0.9867 | 0.002477 | 0.097547 | no |
| DDX11-AS1 | ENST00000500527.1 | 195 | 0.9649 | 0.133397 | 0.099794 | no |
