# Supplementary material for: Four Autophagy-Related lncRNAs Predict the Prognosis of HCC through Coexpression and ceRNA Mechanism
Source: Biomed Res Int. 2020 Oct 9;2020:3801748. doi: 10.1155/2020/3801748 (PMC7568797; doi:10.1155/2020/3801748)
Supplement: Supplementary 3 — Table S3: K-M and univariate Cox regression analyses of RNAs for OS of 370 HCC patients. [file 3801748.f3.docx]

**Table S3.** K-M and Univariate Cox regression analyses of RNAs for OS of 370 HCC patients

| **RNA** | **Mean±SEM** | **KM** | **B** | **Wald** | **HR** | **95%CI** | ***p-*Value** |
| --- | --- | --- | --- | --- | --- | --- | --- |
| miR-495-3p | 64.66±8.073 | 0.522 | -0.001 | 1.068 | 0.999 | 0.997 - 1.001 | 0.301 |
| DLC1 | 1444.1±50.4 | **0.016** | 0.000 | 4.608 | 1.000 | 1.000 - 1.000 | **0.032** |
| miR-515-5p | 12.44±3.991 | 0.459 | 0.000 | 0.070 | 1.000 | 0.998 - 1.003 | 0.791 |
| DAPK2 | 589.0±30.14 | 0.871 | 0.000 | 2.260 | 1.000 | 0.999 - 1.000 | 0.133 |

Bold numbers indicate significance at ≤ 0.05.
